# Pandemic (H1N1) 2009 Virus on Commercial Swine Farm, Thailand

**DOI:** 10.3201/eid1610.100665

**Published:** 2010-10

**Authors:** Donruethai Sreta, Siriporn Tantawet, Suparlark N. Na Ayudhya, Aunyaratana Thontiravong, Manoosak Wongphatcharachai, Jiradej Lapkuntod, Napawan Bunpapong, Ranida Tuanudom, Sanipa Suradhat, Linda Vimolket, Yong Poovorawan, Roongroje Thanawongnuwech, Alongkorn Amonsin, Pravina Kitikoon

**Affiliations:** Author affiliations: Chulalongkorn University, Bangkok, Thailand (D. Sreta, S.N. Na Ayudhya, A. Thontiravong, M. Wongphatcharachai, J. Lapkuntod, N. Bunpapong, R. Tuanudom, S. Suradhat, L. Vimolket, Y. Poovorawan, R. Thanawongnuwech, A. Amonsin, P. Kitikoon);; Mahidol University, Nakhon Pathom, Thailand (S. Tantawet)

**Keywords:** Pandemic (H1N1) 2009 virus, swine influenza virus, influenza, co-infection, Thailand, viruses, pigs, dispatch

## Abstract

A swine influenza outbreak occurred on a commercial pig farm in Thailand. Outbreak investigation indicated that pigs were co-infected with pandemic (H1N1) 2009 virus and seasonal influenza (H1N1) viruses. No evidence of gene reassortment or pig-to-human transmission of pandemic (H1N1) 2009 virus was found during the outbreak.

In April 2009, a novel swine origin influenza A (H1N1) virus, now referred to as pandemic (H1N1) 2009 virus, emerged in humans in Mexico and the United States and spread worldwide (*1*). In May 2009, pandemic (H1N1) 2009 was confirmed in 2 patients in Thailand who had a history of travel to Mexico. Shortly after the emergence of this virus, reports of transmission from humans to pigs on pig farms were documented (*2**,**3*). Human-to-pig transmission of this virus was reported in Thailand on December 17, 2009 (www.dld.go.th/dcontrol/Alert/Ah1n1/H1N1%20update22_12_2009.pdf). Pigs showed mild respiratory signs; only 1 pandemic (H1N1) 2009 virus was isolated from 80 nasal swab specimens.

Swine influenza virus (SIV) was reported in Thailand in 1981 (*4*). All 3 subtypes (H1N1, H3N2, and H1N2) of this virus are circulating in Thailand (*5*). A recent pathogenesis study demonstrated that subtype H1N1 induces typical SIV-like illness and slightly more severe gross lesions than illness induced by subtype H3N2 (*6*). Genetic data indicate that SIV (H1N1) in Thailand differs from pandemic (H1N1) 2009 virus. SIV (H1N1) in Thailand contains surface proteins of influenza viruses from North America and Eurasia, which are also found in pandemic (H1N1) 2009 virus; SIV (H1N1) in Thailand contains internal proteins of viruses from Eurasia; and pandemic (H1N1) 2009 viruses contain swine, human, and avian virus gene segments (*5**,**7*).

We report an outbreak of infection with pandemic (H1N1) 2009 virus during November 2009–March 2010 on a commercial pig farm in Thailand. The outbreak presumably resulted from human-to-pig transmission because 1 of the workers on this farm had influenza-like clinical signs at the beginning of the outbreak. Infection in this worker was not confirmed because he quit his job on the farm after the start of the outbreak and could not be located.

## The Study

In early November 2009, a small commercial pig farm in central Thailand reported respiratory problems in pigs (morbidity rate 50%, mortality rate 10%) in nursery pigs. The farm contained 3,235 pigs (700 sows, 35 boars, 1,000 piglets, 1,000 nursery pigs, and 500 finishing pigs). It has a conventional open-house production system in which both sides of the unit have natural air flow ventilation. The farm also has continuous nursery herd flow in which new pigs are continuously added when they are old enough. This process results in pigs of different ages being in the same unit. Sick pigs had clinical signs (fever, cough, nasal discharge, edematous eyelids, and conjunctivitis) of infection.

Nasal swabs from 20 nursery pigs (4–9 weeks of age) were submitted to Chulalongkorn University Veterinary Diagnostic Laboratory. All samples were positive for porcine circovirus type 2 and porcine reproductive and respiratory syndrome virus (these viruses are major causes of swine respiratory disease), and 2 samples were positive for influenza A virus by reverse transcription–PCR (RT-PCR) with primers for each specific pathogen (*8**–**10*).

Because respiratory problems in nursery pigs continued, nasal swabs specimens from 20 nursery pigs and finishing pigs, gilts (young females), and sows (10 per group) with clinical signs were submitted to the diagnostic laboratory by the end of December 2009. Two samples from nursery pigs were positive for influenza virus A (H1N1) by multiplex RT-PCR (*11*). Both samples were subjected to virus isolation in MDCK cells (*12*) and designated RA20 and RA29 (Table 1). Genome characterization identified RA20 as SIV and RA29 as pandemic (H1N1) 2009 virus (Table 2; Figure). SIV-positive nasal swabs obtained in November were then characterized. Results showed that isolates RA4 and RA9 were pandemic (H1N1) 2009 virus, which indicated that pigs on the farm were infected with this virus.

**Table 1:** Influenza (H1N1) viruses studied, Thailand*

Influenza (H1N1) virus isolate	Collection date	Identification	Study designation	GenBank accession no. (gene segment 1–8)
A/sw/Thailand/CU-RA4/2009	2009 Nov 6	Pandemic (H1N1)2009	RA4	CY062305–CY062312
A/sw/Thailand/CU-RA9/2009	2009 Nov 6	Pandemic (H1N1) 2009	RA9	CY062321–CY062328
A/sw/Thailand/CU-RA20/2009	2009 Dec 26	Thai SIV	RA20	CY062281–CY062288
A/sw/Thailand/CU-RA29/2009	2009 Dec 26	Pandemic (H1N1)2009	RA29	CY062297–CY062304
A/sw/Thailand/CU-RA114/2010	2010 Jan 17	Pandemic (H1N1)2009	RA114	CY062265–CY062272
A/sw/Thailand/CU-RA204/2010	2010 Jan 17	Thai SIV	RA204	CY062289–CY062296
A/sw/Thailand/CU-RA15/2010	2010 Jan 30	Pandemic (H1N1) 2009	RA15	CY062273–CY062280
A/sw/Thailand/CU-RA75/2010	2010 Jan 30	Pandemic (H1N1) 2009	RA75	CY062313–CY062320

*SIV, swine influenza virus.

**Table 2:** Gene origin and percent homology of SIV RNA segments compared with pandemic (H1N1) 2009 virus, Thailand*

Influenza (H1N1) virus	PB2 (1–2229)†	PA (1–2153)†	NA (1–1347)†	M (1–982)†	HA (1–1698)†	NS (1–778)†	NP (1–1443)†	PB1 (1–2153)†
Pandemic (H1N1) 2009‡	Avian TRIG		Eurasian swine		Classical swine			Human TRIG
SIV from Thailand§	Eurasian swine				Classical swine		Eurasian swine	
RA4	Avian TRIG		Eurasian swine		Classical swine			Human TRIG
RA9	Avian TRIG		Eurasian swine		Classical swine			Human TRIG
RA20¶	Eurasian swine				Classical swine		Eurasian swine	
	83.1%	85.1%	89.5%	94.2%	86.4%	90.8%	82.4%	85.1%
RA29	Avian TRIG		Eurasian swine		Classical swine			Human TRIG
RA114	Avian TRIG		Eurasian swine		Classical swine			Human TRIG
RA204¶	Eurasian swine				Classical swine		Eurasian swine	
	83.2%	85.2%	89.5%	94.2%	86.8%	90.8%	82.3%	85.1%
RA15	Avian TRIG		Eurasian swine		Classical swine			Human TRIG
RA75	Avian TRIG		Eurasian swine		Classical swine			Human TRIG

*All swine influenza virus (SIV) isolates except RA20 and RA204 have >99% homology with corresponding genes of A/Nonthaburi/102/2009. PB, polymerase B; PA, polymerase A; NA, neuraminidase; M, matrix; HA, hemagglutinin; NS, nonstructural; NP, nucleoprotein; TRIG, triple reassorted internal gene. †Nucleotide positions compared. ‡A/Nonthaburi/102/2009 (H1N1) virus. §A/sw/Ratchaburi/NIAH1481/2000 (H1N1) virus. ¶Percent homology of compared sequences with those of corresponding genes of A/Nonthaburi/102/2009.

Pandemic (H1N1) 2009 investigations on the farm included clinical surveillance and sample collection from sick and contact pigs and close monitoring of swine workers and farm pets for influenza-like illness. Nasal swab specimens were obtained from pigs on January 17, 2010, January 30, 2010, and March 9, 2010. Because initial laboratory findings indicated that the outbreak involved the nursery herd, weaned pigs were moved to a separate site on the farm to control disease in the nursery pigs. Following Food and Agriculture Organization (www.fao.org) sample collection recommendations, we obtained 20 nasal swabs from pigs with SIV-like illness. In addition, nasal swab specimens (n = 10 per group) were collected from gilts, sows, and finishing pigs to test for pandemic (H1N1) 2009 virus, although no clinical signs were observed in any pigs from these age groups. All SIV-positive samples were subjected to virus isolation (*12*), virus subtyping by multiplex RT-PCR (*11*), and whole genome sequencing of subtype H1N1 viruses (*13*). Of 175 samples obtained during December 26, 2009–March 9, 2010, fifteen swab specimens from nursery pigs with clinical signs were positive for influenza (H1N1) 2009 virus; 8 viruses were characterized. No other SIV subtypes were found. On March 9, ≈1 month after implementing the change in handling of pigs, no pigs showed respiratory signs and 34 nasal swab specimens were negative for influenza virus.

**Figure A1:**
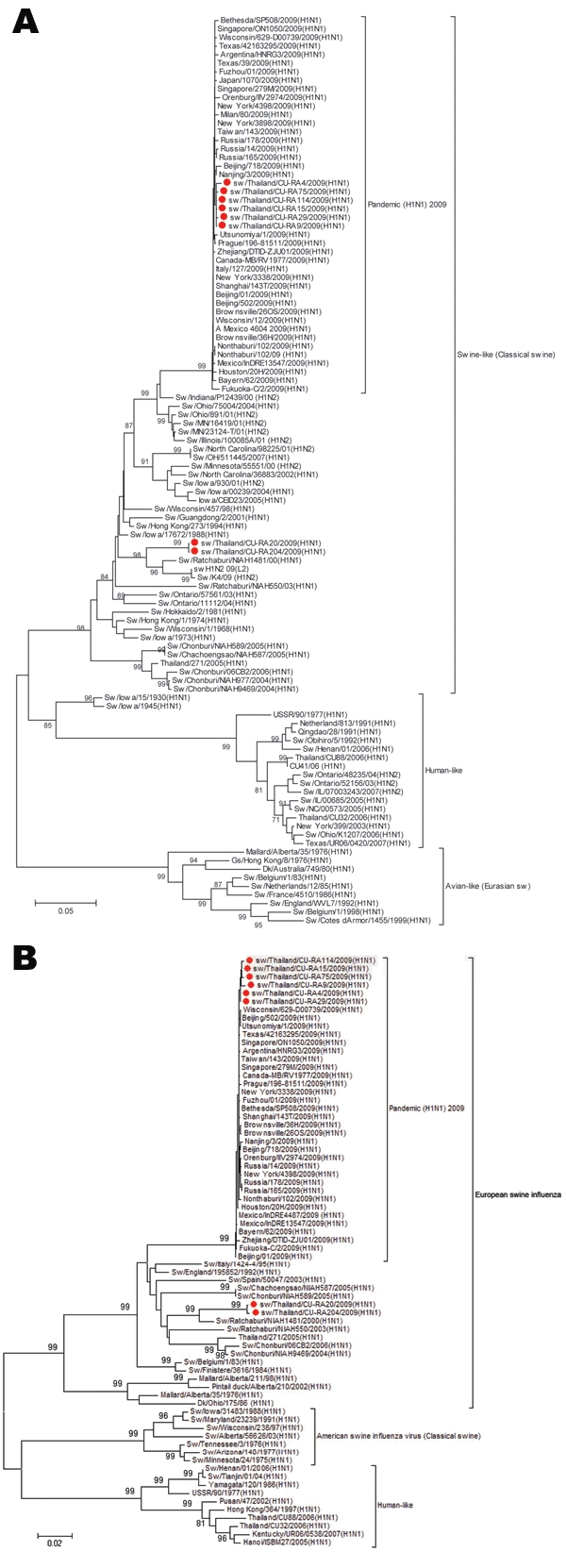
Phylogenetic trees of A) hemagglutinin (HA) and B) neuramindase (NA) genes of swine influenza viruses, Thailand. Red symbols indicate viruses isolated in this study. Scale bars indicate nucleotide substitutions per site.

Gene sequences were compared for corresponding genes of other influenza virus strains obtained from GenBank by using the MegAlign program (DNASTAR, Madison, WI, USA). Phylogenetic trees were constructed by using MEGA4 (www.megasoftware.net/) and the neighbor-joining method with 1,000 bootstrap replicates. Whole genome analysis showed that contemporary SIV (H1N1) and pandemic (H1N1) 2009 virus were concurrently circulating in the nursery herd (Table 2; Figure). On the basis of virus hemagglutinin 1 gene grouping (*14*), our findings show that newly isolated SIV (H1N1) from Thailand are grouped in the classical swine cluster with other SIV (H1N1) isolates (Figure A1). There was no evidence of gene reassortment between SIV (H1N1) and pandemic (H1N1)2009 virus during the investigation (Table 2).

To test for evidence of pandemic (H1N1) 2009 virus interspecies transmission, we obtained serum samples on January 17, 2010, from 40 pigs in 8 age groups (5/group), 15 workers, and 4 farm pets (3 dogs and 1 cat). Samples were subjected to hemagglutination-inhibition (HI) testing with SIV (H1N1) and pandemic (H1N1) 2009 virus antigens (*12*).

Control rabbit antibodies against SIV (H1N1) viruses did not cross-react with pandemic (H1N1) 2009 virus. Serologic results showed that only 2 (9.5%) of 21 test samples from the nursery group had positive HI titers for pandemic (H1N1) 2009 virus and 8 (38%) of 21 had positive HI titers for SIV (H1N1) virus. For pigs in other age groups, 11 (55%) of 20 had antibodies against pandemic (H1N1) 2009 virus and 14 (70%) of 20 had antibodies against SIV (H1N1) by HI test. No human cases of co-infection were observed. We found no evidence of pandemic (H1N1) 2009 virus interspecies transmission from pigs to humans or to farm pets.

## Conclusions

Consistent with findings of previous reports (*2**,**3*), our findings demonstrate that young pigs are susceptible to infection with pandemic (H1N1) 2009 virus. Infection in pigs substantiates the hypothesis that the clinical outcome caused by infection with pandemic (H1N1) 2009 virus differs from that of infection with SIV (H1N1), which currently circulates in pigs in Thailand. Serologic results demonstrated that uninfected populations are susceptible to infection with pandemic (H1N1) 2009 virus. Results of genome analysis did not show gene reassortment between the 2 different influenza (H1N1) viruses. However, a previous report showed that reassortment of influenza virus genes occurs in pigs (*15*). Continued monitoring, characterization of SIVs, and serologic surveillance of pigs are necessary for future influenza pandemic preparedness.

## Figures and Tables

**Figure:**
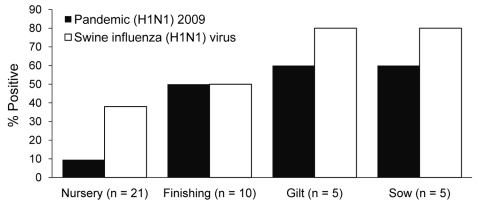
Percent of pigs with antibodies against pandemic (H1N1) 2009 virus and swine influenza virus (H1N1) detected by hemagglutination-inhibition test, by pig type, Thailand. Serum samples were obtained from pigs of different ages in January 2010. Samples were positive when titer was >40.

## References

[1] Novel Swine-Origin Infleunza A (H1N1)Virus Investigation Team, Dawood FS, Jain S, Finelli L, Shaw MW, Lindstrom S, Garten RJ, Emergence of a novel swine-origin influenza A (H1N1) virus in humans. N Engl J Med. 2009;360:2605–15. 10.1056/NEJMoa0903810 19423869

[2] Pereda A, Cappuccio J, Quiroga MA, Baumeister E, Insarralde L, Ibar M, Pandemic (H1N1) 2009 outbreak on pig farm, Argentina. Emerg Infect Dis. 2010;16:304–7. 20113566 10.3201/eid1602.091230 PMC2958022

[3] Pasma T, Joseph T. Pandemic (H1N1) 2009 infection in swine herds, Manitoba, Canada. Emerg Infect Dis. 2010;16:706–8. 20350394 10.3201/eid1604.091636 PMC3321968

[4] Kanai C, Suwicha K, Nadhirat S, Nerome K, Nakayama M, Oya A. Isolation and serological characterization of influenza A virus from a pig in Thailand. Jpn J Med Sci Biol. 1981;34:175–8. 7311111 10.7883/yoken1952.34.175

[5] Takemae N, Parchariyanon S, Damrongwatanapokin S, Uchida Y, Ruttanapumma R, Watanabe C, Genetic diversity of swine influenza viruses isolated from pigs during 2000 to 2005 in Thailand. Influenza Other Respir Viruses. 2008;2:181–9. 10.1111/j.1750-2659.2008.00062.x 19453423 PMC4941901

[6] Sreta D, Kedkovid R, Tuamsang S, Kitikoon P, Thanawongnuwech R. Pathogenesis of swine influenza virus (Thai isolates) in weanling pigs: an experimental trial. Virol J. 2009;6:34. 10.1186/1743-422X-6-34 19317918 PMC2678088

[7] Kingsford C, Nagarajan N, Salzberg SL. 2009 Swine-origin influenza A (H1N1) resembles previous influenza isolates. PLoS ONE. 2009;4:e6402. 10.1371/journal.pone.0006402 19636415 PMC2712239

[8] Payungporn S, Phakdeewirot P, Chutinimitkul S, Theamboonlers A, Keawcharoen J, Oraveerakul K, Single-step multiplex reverse transcription–polymerase chain reaction (RT-PCR) for influenza A virus subtype H5N1 detection. Viral Immunol. 2004;17:588–93. 10.1089/vim.2004.17.588 15671756

[9] Kim J, Chae C. A comparison of virus isolation, polymerase chain reaction, immunohistochemistry, and in situ hybridization for the detection of porcine circovirus 2 and porcine parvovirus in experimentally and naturally coinfected pigs. J Vet Diagn Invest. 2004;16:45–50. 10.1177/104063870401600108 14974846

[10] Thanawongnuwech R, Amonsin A, Tatsanakit A, Damrongwatanapokin S. Genetics and geographical variation of porcine reproductive and respiratory syndrome virus (PRRSV) in Thailand. Vet Microbiol. 2004;101:9–21. 10.1016/j.vetmic.2004.03.005 15201029

[11] Choi YK, Goyal SM, Kang SW, Farnham MW, Joo HS. Detection and subtyping of swine influenza H1N1, H1N2 and H3N2 viruses in clinical samples using two multiplex RT-PCR assays. J Virol Methods. 2002;102:53–9. 10.1016/S0166-0934(01)00442-6 11879692

[12] Kitikoon P, Nilubol D, Erickson BJ, Janke BH, Hoover TC, Sornsen SA, The immune response and maternal antibody interference to a heterologous H1N1 swine influenza virus infection following vaccination. Vet Immunol Immunopathol. 2006;112:117–28. 10.1016/j.vetimm.2006.02.008 16621020

[13] Hoffmann E, Stech J, Guan Y, Webster RG, Perez DR. Universal primer set for the full-length amplification of all influenza A viruses. Arch Virol. 2001;146:2275–89. 10.1007/s007050170002 11811679

[14] Vincent AL, Ma W, Lager KM, Gramer MR, Richt JA, Janke BH. Characterization of a newly emerged genetic cluster of H1N1 and H1N2 swine influenza virus in the United States. Virus Genes. 2009; Jul 14. [Epub ahead of print]. 10.1007/s11262-009-0386-6 19597980

[15] Castrucci MR, Donatelli I, Sidoli L, Barigazzi G, Kawaoka Y, Webster RG. Genetic reassortment between avian and human influenza A viruses in Italian pigs. Virology. 1993;193:503–6. 10.1006/viro.1993.1155 8438586

